# Policing practices as a structural determinant for HIV among sex workers: a systematic review of empirical findings

**DOI:** 10.7448/IAS.19.4.20883

**Published:** 2016-07-18

**Authors:** Katherine HA Footer, Bradley E Silberzahn, Kayla N Tormohlen, Susan G Sherman

**Affiliations:** 1 Department of Epidemiology, Johns Hopkins Bloomberg School of Public Health, Baltimore, MD, USA; 2 Center for Public Health and Human Rights, Johns Hopkins Bloomberg School of Public Health, Baltimore, MD, USA

**Keywords:** systematic review, HIV, police, sex work, arrest, STI, measurement

## Abstract

**Introduction:**

Sex workers are disproportionately infected with HIV worldwide. Significant focus has been placed on understanding the structural determinants of HIV and designing related interventions. Although there is growing international evidence that policing is an important structural HIV determinant among sex workers, the evidence has not been systematically reviewed.

**Methods:**

We conducted a systematic review of quantitative studies to examine the effects of policing on HIV and STI infection and HIV-related outcomes (condom use; syringe use; number of clients; HIV/STI testing and access) among cis and trans women sex workers. Databases included PubMed, Embase, Scopus, Sociological Abstracts, Popline, Global Health (OVID), Web of Science, IBSS, IndMed and WHOLIS. We searched for studies that included police practices as an exposure for HIV or STI infection or HIV-related outcomes.

**Results:**

Of the 137 peer-reviewed articles identified for full text review, 14 were included, representing sex workers' experiences with police across five settings. Arrest was the most commonly explored measure with between 6 and 45% of sex workers reporting having ever been arrested. Sexual coercion was observed between 3 and 37% of the time and police extortion between 12 and 28% across studies. Half the studies used a single measure to capture police behaviours. Studies predominantly focused on “extra-legal policing practices,” with insufficient attention to the role of “legal enforcement activities”. All studies found an association between police behaviours and HIV or STI infection, or a related risk behaviour.

**Conclusions:**

The review points to a small body of evidence that confirms policing practices as an important structural HIV determinant for sex workers, but studies lack generalizability with respect to identifying those police behaviours most relevant to women's HIV risk environment.

## Introduction

Globally, sex workers, defined as those who exchange sex for money or other goods have a disproportionate burden of HIV [1–7]. For over a decade, public health and social science research has sought to more clearly link HIV to the social production of behavioural risk and transmission [8–11]. The conceptual shift from individual-focused HIV prevention (e.g. behaviour change, communication) to environmental-structural interventions emerged as far back as the 1990s among female sex workers (FSWs) [12–14]. Recent research places these intervention efforts in context, highlighting a lack of empirical work to delineate the epidemiology of structural risk factors and HIV among FSWs [15]. An assortment of theoretically informed frameworks exist to elucidate the dynamic socio-economic production of HIV infection across levels, from the macro (e.g. stigmatization of sex work, laws governing possession of drugs) to the micro (e.g. local policing practices, access to drug treatment programs), and how such pathways relate to interpersonal, individual and biological factors [15–17]. A recent call to action to address the HIV response to sex work included a focus on structural reforms and interventions [18]. It is fundamental to the scientific credibility of prioritizing structural interventions to ensure that the measurement of structural determinants (e.g. stigma, local policing practices) is properly operationalized, and if possible, independently associated with specific HIV-related outcomes (e.g. condom use, HIV or STI infection). Law is increasingly viewed as a significant HIV determinant, but one that is a “complex phenomenon” in its own right [19], operating at different structural levels to influence HIV risk behaviours and acquisition (e.g. macro-level laws, meso-level legal policies and micro-level policing practices.)

### Laws and policies that influence HIV risk

Criminal laws related to sex work have been linked to fewer public health benefits, and yet it remains the dominant global approach [20–23]. Across different settings punitive laws take a variety of forms, from criminalizing the transaction itself to prohibiting acts related to sex work, such as solicitation, being found in a brothel or communicating for the purposes of prostitution [24,25]. Other laws of more general application are also frequently employed (e.g. loitering, public indecency, disorderly behaviour offences). Modelling undertaken by Shannon *et al*. suggests the decriminalization of sex work could have one of the greatest effects on the course of the HIV epidemic across settings with concentrated or generalized HIV epidemics [26]. Potentially, sex workers who inject drugs risk exposure to both drug- and sex worker-related laws. Research on drug-related law enforcement practices and its positive association with HIV prevalence among people who inject drugs points to the limited deterrent effect of such practices, and the parallel need for reforms to drug laws and policies [27–30].

### Policing practices and sex workers' HIV risk

The transformative process by which the law “on the books,” e.g. statutes, regulations and court decisions, is manifested in the “street level” practices of the police is of central importance [19]. Legal scholars have long demonstrated the gap between written law and law enforcement [19,31]. In settings in which sex work is criminalized, the police are able to exercise considerable discretion in shaping law enforcement activities regarding sex workers. Policing practices can directly and indirectly affect sex workers HIV-related behaviours. A key example of a direct effect is the confiscation or destruction of condoms, which can prompt unprotected sex, as has been documented worldwide [32–36]. In the case of sex workers who also inject drugs, the confiscation of drugs and injecting equipment by police in the absence of arrest is associated with HIV infection [37–42]. Policing practices that indirectly affect sex workers’ HIV risk behaviours include periodic crackdowns or moving women off corners to address community disquiet [34,43–47]. These strategies can displace sex workers to unfamiliar areas, increase their potential exposure to violence and severely limit their negotiating power with clients [43,47–51].

Egregious abuses of police power are receiving particular attention in the literature. The normalisation of sexual violence as a tool of law enforcement has been documented worldwide [52], including forced unprotected sex [53–57] and coercive sex (e.g. sex in exchange for no arrest) [36,55,58–65]. Other rights violations that sex workers suffer at the hands of the police include verbal harassment and humiliation [64,66–69]; financial extortion to avoid arrest [4,54,57,61–63,70,71]; intimidation or physical violence [54,55,64,72,73] and arbitrary arrest or detention, frequently accompanied by illegal searches and physical violence [50,56,58,70,74].

### Previous reviews

A systematic review of violence against sex workers by Deering *et al*. [75] identified policing practice (either lawful or unlawful) as a key correlate. Another systematic review by Shannon *et al*. [26] reviewed data for HIV prevalence and incidence, condom use and structural determinants among FSWs. Most of the 149 articles included in the review addressed other structural determinants, with seven articles measuring policing practices as a structural vulnerability a priori within women's work environment. The authors concluded that police abuses and law enforcement strategies are key barriers to HIV prevention efforts among FSWs worldwide. In addition to these systematic reviews, two other non-systematic reviews of the evidence have provided important contributions to the field. Decker *et al*. placed a spotlight on police abuses as a contributing factor to sex workers’ experience of human rights violations [52], while Tenni *et al*. reviewed the scope and opportunities for interventions that address policing practices towards sex workers [76].

### Current review

The goal of the current systematic review is to provide a focus on how quantitative research has operationalized the measurement of law enforcement practices as a structural determinant of HIV for women (including transgender) sex workers. The review builds on previous work by critiquing the strength of the evidence to support the legal environment's role as a structural HIV determinant with respect to micro-level policing practices, situated as they are in a broader and more complex legal environment. An important distinction in this review is the inclusion of search terms for cis and trans women sex workers. The review also includes a broader range of HIV-related outcomes, and seeks to provide a more critical review of methodological issues in interpreting findings in the existing quantitative literature.

## Methods

### Search strategy and selection criteria

We followed PRISMA guidelines in conducting the systematic review. Subject headings and associated terms were initially developed and tested in PubMed and adapted for other database specific categories (see Supplementary file 1 for full list of search terms). The search strategy involved two extensive search components for each database, and was developed to reliably capture: (1) police enforcement strategies, both legal and illegal; and (2) terms relevant to our population of interest (i.e. sex workers). A comprehensive review of all major databases was undertaken on 18 September 2015 (BS). Databases searched for this review were PubMed, Embase, Scopus, Sociological Abstracts, Popline, Global Health (OVID), PAIS International, Criminal Justice Abstracts, Web of Science, IBSS, WHO Regional Databases, IndMed and WHOLIS. Ancestry searches were also conducted for references and the relevant citations to all studies.

We included articles that examined police practices as an exposure variable for HIV and/or STI infection or HIV-related outcomes. The studies under consideration were required to use quantitative univariate or multivariable methods and to have been published in a peer-reviewed journal. Primary outcomes of interest were HIV infection, STI infection and STI symptoms. Secondary outcomes were HIV/STI testing and access, number of clients, condom use and syringe use. Studies were included for cis and trans women who exchanged sex for money or other goods (i.e. sex work); we excluded studies focused exclusively on male sex workers or adolescents, and studies only including indoor work environments (e.g. brothels, massage parlours, hotels, saunas). Based on a signal to noise ratio of each year searched, we limited our search to articles published between 1 January 2006 and 18 September 2015. Articles were limited to those published in the English language, but were not limited by either quantitative study design or setting.

### Screening and data abstraction

Article citations were organized and uploaded to Endnote, and subsequently reviewed using Rayyan, a web application for exploring and filtering systematic review searches [77]. The title and abstracts of all publications were originally screened by two independent reviewers (BS and KT) to retain those that clearly met the inclusion criteria, or for whom the full text of the article had to be reviewed before a final determination on inclusion could be made. Two independent reviewers (KF and BS) undertook full text reviews, and any discrepancies as to final inclusion were discussed with the senior reviewer (SS) to reach a consensus as to whether to exclude or include any specific article. Data were abstracted using a standard abstraction form for each study (see Supplementary file 1). Due to the lack of validated quality assessment checklists for cross-sectional studies, the authors undertook a critical appraisal of studies during the data abstraction process [78–81].

As shown in Table 1, we set out the police measures for each study and how frequently they occurred (if reported), along with the primary outcomes of interest (HIV infection, STI infection, STI symptoms) and secondary outcomes (access to HIV services, number of clients, condom use and syringe use). If results from both univariate and multivariate models were presented, we extracted the (adjusted) associations of the multivariate model only.

**Table 1:** Study and intervention characteristics, description of HIV infection and sexually transmitted infection outcomes and police measures

Author, publication date	Country (cities)	Legal status	Study dates	Study design	Sample size, Population of interest	Police measure	% Reporting police measure	Outcome	% With outcome (overall/population with police measure only)	Association (95% CI)
Chen *et al*., 2012 [82]	Mexico (Nuevo Laredo, Ciudad Hidalgo)	Regulated in specific zones but soliciting in public is illegal	2009–2010	Cross-sectional	200 FSWs	Ever arrested	28.6	- Current STI symptoms	16.5/45.5	aOR: 2.3 (1.0, 5.0)
						Arrested in prior year	16.5	- Current STI symptoms	16.5/27.3	OR: 2.2 (0.9, 5.4)
Decker *et al*., 2012 [4]	Russia (Moscow)	Selling sex is illegal in any venue	2005	Cross-sectional	147 FSWs	Sexual coercion	36.6	- Current HIV infection/ STI (any)	31.3/44.2	aOR: 2.5 (1.2, 5.4)
Decker *et al*., 2014 [83]	Russia (Kazan, Krasnoyarsk, Tomsk)	Selling sex is illegal in any venue.	2011	Cross-sectional	754 FSWs	Sexual coercion	3.1	- Current HIV Infection	3.9/13.0	aOR: 3.5 (0.9, 12.9)
						Extortion	28.4	- Current HIV Infection	3.9/5.1	aOR: 1.1 (0.5, 2.2)
Erausquin *et al*., 2011 [70]	India (Rajahmundry)	Organizing commercial sex in any place and soliciting in public is illegal	2009–2010	Cross-sectional	835 FSWs	Sexual coercion	10.9	- Recent STI symptoms - Inconsistent condom use (C) - Accepted more money for sex with no condom (C)	48.5/74.7 24.0/31.9 18.1/28.6	aOR: 3.6 (2.1, 5.9) aOR: 1.5 (0.9, 2.5) aOR: 2.0 (1.2, 3.4)
						Extortion	12.0	- Recent STI symptoms - Inconsistent condom (C) - Accepted more money for sex with no condom (C)	48.5/81.0 24.0/41.0 18.1/34.0	aOR: 5.1 (3.0, 8.7) aOR: 2.4 (1.5, 3.7) aOR: 2.8 (1.7, 4.4)
						Condoms confiscated	7.4	- Recent STI symptoms - Inconsistent condom (C) - Accepted more money for sex with no condom (C)	48.5/74.2 24.0/51.6 18.1/43.6	aOR: 3.1 (1.7, 5.7) aOR: 3.6 (2.1, 6.1) aOR: 4.1 (2.3, 7.2)
						Workplace raid	26.8	- Recent STI symptoms - Inconsistent condom use (C) - Accepted more money for sex with no condom (C)	48.5/71.4 24.0/26.4 18.1/21.9	aOR: 3.7 (2.6, 5.3) aOR: 1.2 (0.8, 1.7) aOR: 1.5 (1.0, 2.3)
						Arrested during prior 6 months	12.0	- Recent STI symptoms - Inconsistent condom use (C) - Accepted more money for sex with no condom (C)	48.5/77.0 24.0/35.0 18.1/31.0	aOR: 3.8 (2.3, 6.2) aOR: 1.8 (1.1, 2.9) aOR: 2.4 (1.4, 3.9)
Erausquin *et al*., 2014 [84]	India (Rajahmundry)	Organizing commercial sex in any place and soliciting in public is illegal	2006–2010	Cross-sectional	1680 FSWs	Sexual coercion	11.1	- Recent STI symptoms - Inconsistent condom use (C) - Accepted more money for sex with no condom (C)	44.3/65.6 34.2/33.9 21.3/33.3	aOR: 2.2 (1.6, 3.1) aOR: 1.2 (0.8, 1.6) aOR: 2.0 (1.4, 2.9)
						Extortion	14.8	- Recent STI symptoms - Inconsistent condom use (C) - Accepted more money for sex with no condom (C)	44.3/64.9 34.2/42.3 21.3/36.3	aOR: 2.4 (1.8, 3.2) aOR: 1.6 (1.2, 2.1) aOR: 2.5 (1.8, 3.3)
						Condoms confiscated	7.6	- Recent STI symptoms - Inconsistent condom (C) - Accepted more money for sex with no condom (C)	44.3/66.4 34.2/43.8 21.3/46.9	aOR: 2.4 (1.6, 3.6) aOR: 1.7 (1.2, 2.5) aOR: 3.8 (2.6, 5.6)
						Workplace raid	36.1	- Recent STI symptoms - Inconsistent condom (C) - Accepted more money for sex with no condom (C)	44.3/58.3 34.2/34.8 21.3/26.5	aOR: 2.2 (1.8, 2.8) aOR: 1.1 (0.9, 1.4) aOR: 1.6 (1.2, 2.0)
						Arrested during prior 6 months	14.5	- Recent STI symptoms - Inconsistent condom use (C) - Accepted more money for sex with no condom (C)	44.3/60.1 34.2/35.8 21.3/28.8	aOR: 1.7 (1.3, 2.3) aOR: 1.2 (0.9, 1.6) aOR: 1.5 (1.1, 2.1)
						Summary measure (1 police interaction)	19.8	- Recent STI symptoms - Inconsistent condom (C) - Accepted more money sex with no condom (C)	44.3/52.1 34.2/29.5 21.3/18.1	aOR: 2.0 (1.5, 2.6) aOR: 0.9 (0.7, 1.2) aOR: 1.0 (0.7, 1.4)
						Summary measure (2 or more police interaction)	22.7	- Recent STI symptoms - Inconsistent condom use (C) - Accepted more money for sex with no condom (C)	44.3/63.1 34.2/38.2 21.3/33.3	aOR: 3.0 (2.3, 3.9) aOR: 1.4 (1.0, 1.8) aOR: 2.4 (1.8, 3.2)
Erickson *et al*., 2015 [85]	Uganda (Gulu)	Selling sex is illegal in any venue	2011–2012	Cross-sectional	400 FSWs	Police presence rushed negotiations	37.3	- Dual contraceptive use (past 6 months)	45.0/31.0	aOR: 0.7 (0.4, 1.0)
						Displacement by police	28.9	- Dual contraceptive use (past 6 months)	45.0/28.0	OR: 0.9 (0.6, 1.4)
Gertler and Shah, 2011 [86]	Ecuador (various)	Regulated indoors. No laws govern street-based sex work but public order offences are used	2003	Cross-sectional	2914 FSWs	Street enforcement	NA	- Current STI (any) - Ever STI Infection - Current herpes infection	NA NA NA	Beta: −0.1^a^ Beta: −0.3^a^ Beta: −0.1^a^
Pando *et al*., 2013 [87]	Argentina (various)	Organizing commercial sex in any place and soliciting in public is illegal	2006–2009	Cross-sectional	1255 FSWs	Ever arrested	45.4	- Current HIV infection - Current syphilis infection - Inconsistent condom use (N-C) - Inconsistent condom (C)	2.0/3.2 22.4/29.5 82.4/84.4 11.4/14.7	aOR: 1.8 (1.1, 3.0) aOR: 1.5 (1.2, 1.7) aOR: 1.0 (0.8, 1.3) aOR: 1.1 (0.9, 1.4)
Pitpitan *et al*., 2015 [88]	Mexico (Ciudad Juarez)	Regulated in specific zones but soliciting in public is illegal	2010	Randomized control trial	213 FSWs–IDUs	Syringe confiscation	NA	- Safe injection	NA	aOR: 0.6 (0.4, 1.0)
Qiao *et al*., 2014 [89]	China (Guangxi)	Selling sex is illegal in any venue	2011	Cross-sectional	794 FSWs	Ever arrested	5.7	- Consistent condom use (C) - Access HIV testing - Access HIV prevention services	<39/NA <50/NA 73/NA	OR: 0.8 (0.4, 1.5) aOR: 2.8 (1.2, 6.2) aOR: 4.6 (0.9, 23.3)
Shannon *et al*., 2009 [51]	Canada (Vancouver)	Organizing commercial sex in any place and soliciting in public is illegal^b^	2006	Cross-sectional	205 FSWs–TWS	Displacement by police	44.4	- Pressured into unprotected sexual intercourse with client in last 6 months	24.9/38.4	aOR: 3.1 (1.4, 7.4)
						Zoning restriction following previous charge	8.7	- Pressured into unprotected sexual intercourse with client in last 6 months	24.9/50.0	aOR: 3.4 (1.2, 9.4)
Strathdee *et al*., 2011 [90]	Mexico (Cuidad Juarez, Tijuana)	Regulated in specific zones but soliciting in public is illegal	2008–2009	Cross-sectional	620 FSWs–IDUs	Syringe confiscation	29.0	- Current HIV infection	5.3/8.1	aOR: 2.4 (1.2, 5.0)
Strathdee *et al*., 2013 [5]	Mexico (Cuidad Juarez)	Regulated in specific zones but soliciting in public is illegal	2008–2010	Randomized control trial	300 FSWs–IDUs	Arrested during prior 6 months	49.0	- HIV/STI incidence^d^	67.4/NA	NA
	Mexico (Tijuana)		2008–2010	Randomized control trial	284 FSWs–IDUs	Arrested during prior 6 months	40.9	- HIV/STI incidence^d^	44.3/NA	aRR: 2.7 (1.4,5.2)^c^
Zhang *et al*., 2013 [91]	China (Guangxi)	Selling sex is illegal in any venue	2011	Cross-sectional	720 FSWs	Ever arrested	NA	- Unprotected sex in the last sex act	NA	aOR: 2.6 (1.4, 4.7)

a Population-level estimates across communities of adjusted beta-coefficient using linear regression

b The law changed in 2014 to decriminalize but this research was conducted prior to the change

c Population-level estimates from Poisson regression across different risk groups

d HIV/STI incidence density over 12 months across four different intervention groups.

FSW=cis female sex worker; TWS=transgender woman sex worker; IDU=injection drug use; C=client; N-C=non-commercial partner; OR=odds ratio; aOR=adjusted odds ratio; aRR=adjusted relative risk; Beta=beta-coefficient from linear regression.

### Data synthesis

**Figure 1:**
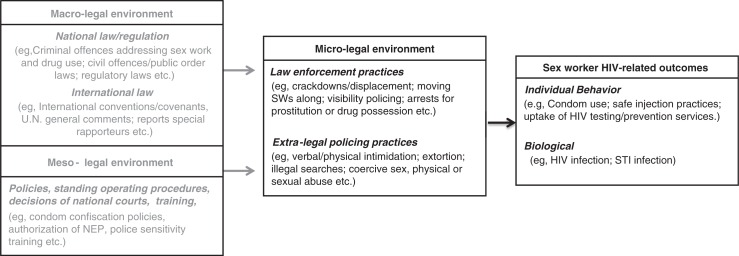
Framework of law as a structural determinant for health outcomes of sex workers, adapted from Burris and colleagues [19].

Due to the diversity in policing determinants and HIV outcomes, a meta-analysis was not conducted. Instead, to guide our review, we have adapted a conceptual model that provides a legal determinants framework of HIV-related outcomes for sex workers (Figure 1), and draw on previous ecological [19] and risk environment frameworks [16,17]. The framework highlights how macro-level laws and meso-level policies are critical influences on sex workers’ HIV risk environment. However, it is at the micro-level of policing practices that laws and policies become interpreted in the everyday decision-making of police officers. The framework draws a distinction between enforcement practices within the law and those outside of it, which we define as “extra-legal policing practices” [30]. The latter represent behaviours that sit along a spectrum from misconduct to illegal behaviours, with human rights violations a prevalent feature in some settings.

## Results

**Figure 2:**
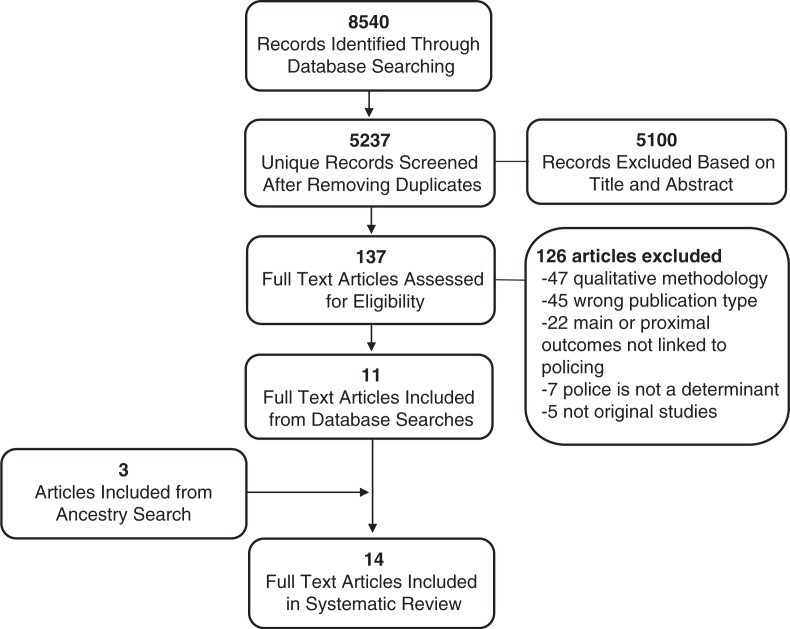
Flowchart of search strategy.

The search process is described in Figure 2, and results are displayed in Table 1. Overall, 14 quantitative studies [4,5,51,70,82–91] were identified that met our inclusion criteria. These papers represent the current empirical evidence that policing practices are a key micro-structural determinant for HIV risk among sex workers.

### Study characteristics

The studies cover a fairly diverse range of geographic regions given the number of studies, but the diversity of countries within regions was small. Seven of the studies were conducted in the Americas. Four were in Mexico, the two from Eastern Europe were both in Russia, one was in the Central East African country of Uganda, four were in Asia with two in India and two in China. Five of the 14 papers were conducted in settings in which selling sex in any venue is illegal [4,83,85,89,91]. For the four studies set in Mexico [5,82,88,90], sex work is regulated in specific zones, but it remains illegal to solicit sex in public. Four of the 14 studies [51,70,84,87] were in settings in which it is illegal to solicit sex in any public place or organize commercial sex in any place. In one study [86], indoor sex work is regulated and while no laws govern street-based sex work, police often employ public order offences [92].

One of the studies considered women inclusive of trans [51], while cis-woman sex workers who inject drugs were considered in two of the 14 papers [5,90]. All of the studies except one included samples of sex workers from multiple sex work environments (e.g. brothels, street, motels). The exception was one study exclusively focused on truck stops as a sex work venue [82]. Only two out of the 14 studies statedthat both over and under 18-year-olds were included in their sample [4,85].

### Study design and measures

Twelve of the papers (86%) used a cross-sectional study design, while two (14%) analyzed the results of the same randomized controlled study [5,88], conducted in the context of an intervention. In nine of the 14 studies (64%) there was a single measure for police behaviour. Considering legal enforcement practices as set out in our conceptual framework, five of the six studies measuring this concept looked at arrest in some form (i.e. any arrest ever, a history of arrest or arrest in the last six months) as the only measure of police practices [5,82,87,89,91]. Only one study defined arrest as arrest for sex worker-related activity [87]. The remaining three single measure studies look at what we term “extra-legal policing practices”. Two measured syringe confiscation in the context of FSWs who inject drugs in settings where syringes can be obtained legally by prescription [88,90], and one looked at sexual coercion by police [4]. The remaining five studies (36%) considered between two and five measures to assess police practices towards sex workers. In two of these studies, police measures were made up of five items [70,84], although both of these used the same data and measures. Those papers containing more than one measure included a mixture of extra-legal policing practices, such as sexual coercion (e.g. sex exchanged for leniency or to avoid arrest or trouble) [70,83,84]; extortion (e.g. money, gifts, bribe) [70,83,84]; and condom confiscation [70,84]. Day-to-day policing strategies aside from arrest included, displacing sex workers and zoning restrictions [51], conducting workplace raids [70,84] and general policing presence [85].

Three papers considered the impact of interventions. The only one that focused on changing police behaviours evaluated the impact of a community-led structural intervention, targeting both FSWs and the police (including providing access to STI treatment, educating officers about HIV and conducting sensitivity meetings between officers and FSWs) [84]. The paper analyzed data from cross-sectional studies conducted over three different time points during the course of the intervention to explore changes in STI symptoms, inconsistent condom use and accepting more money for sex without a condom. The other two papers analyzed the results of a study that used a 2x2 randomized factorial trial in which participants were exposed to a combination of either a 30-minute didactic or interactive intervention focused on safe injection and sex practices [5,88]. One paper evaluated the efficacy of the different interventions on HIV and STI infection incidence, adjusting for the level of “recent arrest” in each intervention group [5]. The other paper looked at the efficacy of the interventions in reducing receptive needle sharing, with police behaviours as moderators (i.e. arrest and syringe confiscation) [88].

### Frequency of police measures

Overall, the studies included in our analysis found that police measures were regularly reported by sex workers (Table 1). A simple average across the most common reported police measures within each study suggests that 34% of sex workers experienced at least one police measure (range: 6–49%). This average suggests that police measures are very common, granting that it is not a meta-analysis and must be evaluated cautiously. Arrest was the most commonly explored measure with between 6 and 45% of sex workers reporting having ever been arrested, similar to the level found in studies that were limited to measuring arrests in the last six months or a year (range: 12–49%). Sexual coercion was observed between 3 and 37% of the time and police extortion between 12 and 28% across studies.

### Study outcomes

We found that police measures were consistently, positively associated with either our primary (HIV infection or STI infection/symptoms) or secondary (HIV/STI testing and access, number of clients, condom use, and syringe use) outcomes of interest. Having ever been arrested was consistently associated with an increased risk of being currently infected with HIV or an STI. The strength of the association ranged between 1.5 and 2.3 times. It was also associated with inconsistent condom use (aOR range: 1.1–2.6). One study did find that arrest was associated with an increased probability of being tested for HIV (aOR: 2.7). Studies that were limited to arrest in the prior six months or a year still found positive associations with HIV and STI incidence (aOR: 2.7), current/recent STI (aOR range: 1.7–3.8) and inconsistent condom use (aOR range: 1.2–2.4). Other police measures were also found to be important. For example, sexual coercion was associated with current or recent HIV or STI infection (aOR range: 2.2–3.6) and inconsistent condom use (aOR range: 1.2–2). Similarly, police extortion was associated with current or recent HIV or STI infection (aOR range: 1.1–5.1) and inconsistent condom use (aOR range: 1.6–2.8). Syringe confiscation was associated with a 2.4 times increased odds of current HIV infection and 0.6 times the odds of safe injection. Finally, displacement by the police was associated with both inconsistent condom use (aOR: 3.1 times) and reduced (although not significantly) dual contraceptive use (aOR: 0.9).

In the 10 studies in which HIV infection or STI infection and symptoms were an outcome, eight found a significant positive association with at least one police behaviour. One notable exception is a study in Ecuador that found street enforcement was negatively associated with STI symptoms that ever occurred or were current. This represents the only study that found that a police measure was significantly associated with a reduction in any of our outcomes of interest. However, the results from this study should be treated with caution because the analyses were conducted at a population level across different communities with the outcome and exposure data coming from different sources. All seven papers that looked at HIV risk behaviours, instead of (or in addition to) HIV infection or STI infection as an outcome, found a significant association with at least one police behaviour. The most commonly measured HIV risk behaviour was inconsistent condom use [51,70,84,87,89].

Of those studies that were interventions [5,84,88], one directly considered the effectiveness of intervening on interactions between sex workers and the police [84]. The study in question reports that both negative interactions between FSWs and police, and the measures of HIV sexual risk, declined over time from early in the intervention to full implementation. Although these findings are important, the authors also report that the strong association between negative police experiences and HIV risk (e.g. STI symptoms, inconsistent condom use) remained stable and in some cases increased. The other intervention studies both involved data from the same injection risk intervention [5,88]. One found that the intervention “buffered” the negative impacts of police behaviours on risky injection practice [88].

## Discussion

This systematic review points to an important but nascent field of quantitative evidence supporting the significance of policing practices as an important structural HIV determinant in the lives of women who sell sex. We found substantial heterogeneity in both the police measures and the health outcomes considered by the different studies. However, despite the wide array of different associations considered, a significant association between the police measure and a HIV- and STI-associated outcome was reported across a wide range of police measures in the vast majority of analyses of the studies. These findings point to the potentially pivotal role that the police have as a structural determinant for HIV in this vulnerable population.

### Methodology

The majority of included studies are cross-sectional, a study design often used for ease and practicality, particularly with respect to hard-to-reach populations, such as sex workers. Consequently, however, temporality and causation are difficult to establish. Ideally, there is a need for more prospective cohort designs, in which longitudinal data will allow for a more rigorous examination of the cumulative effects of negative police interactions on sex workers’ HIV-related outcomes. Aside from the limitations of relying primarily on cross-sectional study designs, other methodological limitations exist within the 14 studies. For example, three of the 10 studies [70,82,84] (30%) analyzed HIV or STIs not on the basis of biological testing, but instead relied on self-reported STI symptoms, which is not a validated proxy for STI prevalence [82]. In addition, four of the 14 studies (29%) either did not discuss the legal status of sex work or did not make clear whether the police practices (e.g. condom confiscation or syringe confiscation) were prohibited in the location where the study was conducted [5,82,88,91]. Because policies and regulations regarding sex work and drug enforcement differ widely by location, all studies analyzing police practices should have a clear understanding and articulation of the study setting's legal environment in the methods. Finally, because directionality can be unclear, we excluded two papers [38,93] in which policing was considered the outcome variable rather than a structural factor shaping HIV risk outcomes. Addressing directionality can be difficult, however, and employing longitudinal studies in changing policing environments could prove invaluable.

### Measurement

Few studies in this review focus on the measurement of police practices; for the most part, police practices were addressed by only one survey item. We found that most studies relied on crude measures of police behaviours, such as arrest for any offence, or ever having been arrested. First, the results could be biased by overestimating the effect on HIV or STI outcomes of policing activities that were unrelated to sex work and/or occurred years before the outcome was measured. Second, arrest alone is a poor measure of law enforcement that overlooks officers’ day-to-day discretionary enforcement activities other than arrest, as well as extra-legal practices towards sex workers. More focus is needed on measuring law enforcement practices, given the suggestion in the results of these few studies that activities carried out in the name of “public safety” could be an important HIV determinant, alongside more overt abuses of police power. With respect to extra-legal police practices, and mirroring the qualitative and grey literature, the broader context of sex workers’ experience of sexual violence includes a strong focus on policing abuses. This is important, but also draws attention to the lack of empirical evidence supporting important findings in the grey literature around other forms of police abuse and rights violations. For example, condom confiscation is reported in multiple settings [32–34], yet only one author, working in one setting, addressed this topic in two papers [70,84]. In line with the two studies in this review that specifically focused on police practices towards sex work [70,84], police measures to capture a range of police interactions that can be collapsed into a summary measure are needed in future studies. Precise measurement is also lacking with respect to STI infection as an outcome. Of the 10 papers measuring STI as an outcome, only half identify the type of STI infection [4,5,86,87,90], limiting the ability to explore associations between policing and individual STIs. Providing additional detail on specific STIs may also allow us to account for factors that may be correlated with particular STIs (e.g. race/ethnicity, age).

### Gaps and opportunities

Despite our findings of a consistent association between police practices and STI/HIV-associated outcomes, important gaps exist. In particular, the need remains for more quantitative studies to provide a stronger evidence base from which to understand the significance of police practices as part of sex workers’ broader physical HIV risk environment. Specifically, only three of the 14 studies set out to explore the association between HIV outcomes and policing [70,84,86]. Instead, policing was typically considered a secondary exposure of interest. Policing practices as the main exposure of interest would give detail to the measurement of police behaviours, and provide an opportunity to establish more robust associations with HIV outcomes. It is of note that we identified only one study that sought to modify police behaviours [84]. Interventions that target police practices are needed, but prioritizing the intervention components that address policing is limited by the lack of empirical evidence about the epidemiology of police enforcement as a structural risk factor for HIV among sex workers.

This paper's data synthesis is guided by a conceptual model that specifically focuses on a legal determinants framework. Other frameworks, such as the Rhodes risk environment framework, may be better suited to capture the broader context and overlapping dimensions of the risk environment, which represents the space in which a variety of factors exogenous to the individual interact to increase the chances of HIV or STI infection [9]. Future research should look to dynamic conceptual frameworks that support a fuller exploration of the interactions between law enforcement and other features of the environment, including methods that account for these complexities. For example, it is notable that virtually all the papers identified in our search operationalize law enforcement towards sex workers as a single individual-level exposure, common across all individuals. Further, only one study considered police activity as a spatially explicit structural determinant of risk [51]. Related research exploring drug-related law enforcement [28,29,94,95] points to the importance of employing geographic methods to capture the complexities of law enforcement strategies, which may differ greatly across locations. Models with location-specific (or other structural) covariates would move beyond over-simplifying the operationalization of law enforcement as a structural determinant for HIV or STI infection in sex worker populations. Attention should be given to broader data sets that allow meaningful comparison of sex workers across a range of different structural determinants. This needs to be coupled with developments in analytical methods that can help disentangle the impact of such different risk factors on HIV or STI incidence. In particular, the development of hierarchical modelling approaches that take into account different aspects of the structural environment offer an important step forward [26].

Given the small group of papers, it is noteworthy that four of the 14 studies were conducted in Mexico [5,82,88,90] and two in Russia [4,83], meaning 43% of the studies are focused in only two regions. There is a need for future research to reflect a greater geographic diversity that is able to capture both consistencies and differences in policing approaches towards sex workers globally. A final gap in this group of studies is the exclusion of women transgender sex workers. Only one of the 14 studies considers “women” inclusive of both cis and transgender women, and none consider just trans women [51]. A frequent justification for not including transgender women within cis women sex worker studies is that their unique individual, interpersonal and structural vulnerabilities require independent research. However, transgender sex workers are recognized as sharing many of the same structural risks, including those concerning the police [26,96,97]. A large proportion of street-based FSWs are also drug users and face the dual risk of negative police interactions and arrest, as highlighted in three of the papers we included [5,88,90]. Police measures that account for the broader range of negative police interactions experienced by those sex workers who use drugs, and transgender women who sell sex are needed.

### Limitations of study

The review's outcomes should be viewed in light of several limitations. Our inclusion criteria were set so as to produce a narrow critique of the quantitative literature around the role of micro-level policing practices as a structural cause or mitigating factor for risk, and the potential median of structural interventions to reduce risk. We acknowledge the larger body of qualitative and grey literature that supports the role of police abuse and enforcement practices in sex workers’ HIV risk environment. However, this review was concerned with critiquing the strength, generalizability and quality of the present quantitative evidence base. We also exclude studies that looked at the association between police practices and sex workers’ experience of violence. Acknowledging the extensive body of literature that supports the relationship [75], our focus was targeted to the influence policing practices have on more proximal HIV risk behaviours and HIV and STI outcomes among sex workers. Violence is more complicated in its association with HIV risk and has correspondingly been treated distinctly in the literature. The authors also recognize the broader legal and socio structural environment that can mitigate or potentiate sex workers’ risk, of which police practices are only one part. However, it is important to critique individual structural determinants and explore the evidence for their place as “key” determinants for sex workers’ HIV risk. We did not include male sex workers, due to their considerable diversity as a population and distinctiveness compared to FSWs [98]. We chose to include transgender women sex workers, not to undermine their more complex constellation of vulnerabilities, but to present women's experience of police as a structural risk factor. However, given that only one paper included transgender sex workers, the findings lack generalizability to this population. Finally, a meta-analysis was not undertaken due to the heterogeneity in risk factors explored by different studies. Despite these limitations, this review offers the most comprehensive analysis to date of policing practices as a mechanism for HIV outcomes among sex worker populations.

## Conclusions

This review supports the broader literature's finding that there is a strong relationship between police practices and sex workers’ HIV risk behaviours and HIV or STI infection. However, the review exposes a relatively small pool of quantitative epidemiological evidence. Of particular significance is the finding that nearly all the papers identified in this review operationalize policing as an individual-level exposure and fail to take account of the complexities of the risk environment in which law enforcement occurs. A pressing need remains for more epidemiology to better measure and more broadly document both legal and extra-legal enforcement practices as mechanisms through which sex workers’ HIV risk is mediated. The science community should work in partnership with sex work communities and key stakeholders (e.g. police, legal service providers, brothel managers) to build evidence-based evaluations that are grounded in sex workers’ experiences with police. However, it is vital to the success of such interventions that researchers continue to work across varied settings to achieve a more nuanced measurement of the role of police practices as a downstream legal determinant of sex workers’ HIV risk environment.

## Supplementary Material

Policing practices as a structural determinant for HIV among sex workers: a systematic review of empirical findings
