# Supplementary material for: Policing practices as a structural determinant for HIV among sex workers: a systematic review of empirical findings
Source: J Int AIDS Soc. 2016 Jul 18;19(4Suppl 3):20883. doi: 10.7448/IAS.19.4.20883 (PMC4951541; doi:10.7448/IAS.19.4.20883)
Supplement: Policing practices as a structural determinant for HIV among sex workers: a systematic review of empirical findings [file JIAS-19-20883-s001.pdf]

Search Strategy

DATABASE	SEARCH STRINGS
**PubMed**<br><br>**Date Searched**: 09/18/2015<br><br>**Number of Results:** 1,066<br>**After removing Duplicates:** 1,057	1. "Police"[Mesh] OR "Law Enforcement"[Mesh] OR police*[tw] OR policing[tw] OR "law enforcement"[tw] OR officer*[tw] OR cop[tw] OR cops[tw] OR structural determinant*[tw] OR structural factor*[tw] OR "Social Control Policies"[Mesh] OR "Criminal Law"[Mesh] OR "social control"[tw] OR "criminal law"[tw] OR "Social Justice"[Mesh] OR "Civil Rights"[Mesh] OR justice[tw] OR civil right*[tw] OR "Human Rights"[Mesh] OR "crime prevention"[tw]<br><br>2. "Sex Workers"[Mesh] OR "Prostitution"[Mesh] OR sex work*[tw] OR sexual work*[tw] OR sexwork*[tw] OR prostitut*[tw] OR commercial sex*[tw] OR transactional sex*[tw] OR trading sex*[tw] OR traded sex*[tw] OR sex transaction*[tw] OR sexual transaction*[tw] OR exchanging sex*[tw] OR exchanged sex*[tw] OR sexual favor*[tw] OR trade sex*[tw] OR exchange sex*[tw]<br>3. #1 AND #2
**Embase**<br><br>**Date Searched:** 09/18/15<br><br>**Number of Results:** 1,107<br>**After removing Duplicates:** 503	1. 'police'/exp OR 'law enforcement'/exp OR 'social control'/exp OR 'criminal law'/exp OR 'criminal justice'/exp OR (police* OR policing OR "law enforcement" OR officer* OR cop OR cops OR "structural determinant" OR "structural determinant" OR "structural factor" OR "structural factors" OR "social control" OR "criminal law" OR justice OR "civil right" OR "civil rights" OR "crime prevention"):ab,ti<br><br>2. 'prostitution'/exp OR 'transactional sex'/exp OR ((sex NEXT/1 work*) OR (sexual NEXT/1 work*) OR sexwork* OR prostitut* OR (commercial NEXT/1

	sex*) OR (transaction* NEXT/1 sex*) OR (trading NEXT/1 sex*) OR (trade* NEXT/1 sex*) OR (sex* NEXT/1 transaction*) OR (exchang* NEXT/1 sex*) OR (sex* NEXT/1 favor*)):ab,ti<br>3. #1 AND #2
**Scopus**<br><br>**Date Searched:** 09/18/2015<br><br>**Number of Results:** 1,479 hits<br>**After removing Duplicates:** 748	1. TITLE-ABS-KEY ((police* OR policing OR "law enforcement" OR officer* OR cop OR cops OR "structural determinant" OR "structural determinant" OR "structural factor" OR "structural factors" OR "social control" OR "criminal law" OR justice OR "civil right" OR "civil rights" OR "crime prevention"))<br><br>2. TITLE-ABS-KEY (((sex W/1 work*) OR (sexual W/1 work*) OR sexwork* OR prostitut* OR (commercial W/1 sex*) OR (transaction* W/1 sex*) OR (trading W/1 sex*) OR (trade* W/1 sex*) OR (sex* W/1 transaction*) OR (exchang* W/1 sex*) OR (sex* W/1 favor*)))<br>3. #1 AND #2
**Sociological Abstracts**<br><br>**Date Searched:** 09/18/2015<br><br>**Number of Results:** 789<br>**After removing Duplicates:** 526	1. SU.EXACT.EXPLODE("Police" OR "Investigations (Law Enforcement)" OR "Law Enforcement" OR "Criminal Law" OR "Social Control" OR "Social Justice" OR "Civil Rights" OR "Human Rights" OR "Womens Rights" OR "Crime Prevention" OR "Criminal Justice" OR "Criminal Justice Policy") OR ti(police* OR policing OR "law enforcement" OR officer* OR cop OR cops OR "structural determinant*" OR "structural factor*" OR "social control" OR "criminal law" OR justice OR "civil right*" OR "crime prevention") OR ab(police* OR policing OR "law enforcement" OR officer* OR cop OR cops OR "structural determinant*" OR "structural factor*" OR "social control" OR "criminal law" OR justice OR "civil right*" OR "crime prevention")

	2. SU.EXACT.EXPLODE("Prostitution") OR ti("sex work*" OR "sexual work*" OR sexwork* OR prostitut* OR "commercial sex*" OR "transactional sex*" OR "trading sex*" OR "traded sex*" OR "sex transaction*" OR "sexual transaction*" OR "exchanging sex*" OR "exchanged sex*" OR "sexual favor*" OR "trade sex*" OR "exchange sex*") OR ab("sex work*" OR "sexual work*" OR sexwork* OR prostitut* OR "commercial sex*" OR "transactional sex*" OR "trading sex*" OR "traded sex*" OR "sex transaction*" OR "sexual transaction*" OR "exchanging sex*" OR "exchanged sex*" OR "sexual favor*" OR "trade sex*" OR "exchange sex*")<br>3. 1 AND 2
**Popline**<br><br>**Date Searched:** 09/18/2015<br><br>**Number of Results:** 633<br>**After removing Duplicates:** 441	1. ("Police" OR policing OR "law enforcement" OR officer* OR cop OR cops OR "structural determinant*" OR "structural factor*" OR "social control" OR "criminal law" OR "Social Justice" OR "Civil Rights" OR justice OR "civil right*" OR "Human Rights" OR "crime prevention") **AND** ("sex work*" OR "sexual work*" OR sexwork* OR prostitut* OR "commercial sex*" OR "transactional sex*" OR "trading sex*" OR "traded sex*" OR "sex transaction*" OR "sexual transaction*" OR "exchanging sex*" OR "exchanged sex*" OR "sexual favor*" OR "trade sex*" OR "exchange sex*")
**Global Health (OVID)**<br><br>**Date Searched:** 09/18/2015<br><br>**Number of Results:** 482 hits<br>**After removing Duplicates:** 274	1. exp law enforcement/ or exp criminal law/ or exp civil rights/ or exp legal rights/ or exp human rights/ or exp legislation/ or exp law/ or (police* or policing or "law enforcement" or officer* or cop or cops or structural determinant* or structural factor* or "social control" or "criminal law" or justice or civil right* or "crime prevention").tw.<br><br>2. exp sex workers/ or exp prostitutes/ or (sex work* or sexual work* or sexwork* or prostitut* or commercial sex* or transactional sex* or trading sex* or traded

	sex* or sex transaction* or sexual transaction* or exchanging sex* or exchanged sex* or sexual favor* or trade sex* or exchange sex*).tw.<br><br>3. 1 AND 2
**PAIS International**<br><br>**Date Searched:** 09/18/2015<br><br>**Number of Results:** 362<br>**After removing Duplicates:** 304	1. SU.EXACT("Police" OR "Law enforcement" OR "Social control" OR "Criminal law" OR "Social justice" OR "Civil rights" OR "Human rights" OR "Criminal justice" OR "Crime prevention") OR ti(police* OR policing OR "law enforcement" OR officer* OR cop OR cops OR structural determinant* OR structural factor* OR "social control" OR "criminal law" OR justice OR civil right* OR "crime prevention") OR ab(police* OR policing OR "law enforcement" OR officer* OR cop OR cops OR structural determinant* OR structural factor* OR "social control" OR "criminal law" OR justice OR civil right* OR "crime prevention")<br><br>2. SU.EXACT("Prostitution") OR ti(sex work* OR sexual work* OR sexwork* OR prostitut* OR commercial sex* OR transactional sex* OR trading sex* OR traded sex* OR sex transaction* OR sexual transaction* OR exchanging sex* OR exchanged sex* OR sexual favor* OR trade sex* OR exchange sex*) OR ab(sex work* OR sexual work* OR sexwork* OR prostitut* OR commercial sex* OR transactional sex* OR trading sex* OR traded sex* OR sex transaction* OR sexual transaction* OR exchanging sex* OR exchanged sex* OR sexual favor* OR trade sex* OR exchange sex*)<br>3. 1 AND 2

**Criminal Justice Abstracts with Full-Text**<br><br>**Date Searched:** 09/18/2015<br><br>**Number of Results:** 926<br>**After removing Duplicates:** 634	1. ZU("police" OR "law enforcement" OR "social control" OR "criminal law" OR "social justice" OR "civil rights" OR "human rights" OR "crime prevention") OR TI (police* OR policing OR "law enforcement" OR officer* OR cop OR cops OR structural determinant* OR structural factor* OR "social control" OR "criminal law" OR justice OR civil right* OR "crime prevention") OR AB (police* OR policing OR "law enforcement" OR officer* OR cop OR cops OR structural determinant* OR structural factor* OR "social control" OR "criminal law" OR justice OR civil right* OR "crime prevention") OR KW (police* OR policing OR "law enforcement" OR officer* OR cop OR cops OR structural determinant* OR structural factor* OR "social control" OR "criminal law" OR justice OR civil right* OR "crime prevention")<br><br>2. ZU("sex workers" OR "prostitution" OR "prostitutes" OR "transactional sex") OR TI(sex work* OR sexual work* OR sexwork* OR prostitut* OR commercial sex* OR transactional sex* OR trading sex* OR traded sex* OR sex transaction* OR sexual transaction* OR exchanging sex* OR exchanged sex* OR sexual favor* OR trade sex* OR exchange sex*) OR AB(sex work* OR sexual work* OR sexwork* OR prostitut* OR commercial sex* OR transactional sex* OR trading sex* OR traded sex* OR sex transaction* OR sexual transaction* OR exchanging sex* OR exchanged sex* OR sexual favor* OR trade sex* OR exchange sex*) OR KW(sex work* OR sexual work* OR sexwork* OR prostitut* OR commercial sex* OR transactional sex* OR trading sex* OR traded sex* OR sex transaction* OR sexual transaction* OR exchanging sex* OR exchanged sex* OR sexual favor* OR trade sex* OR exchange sex*)<br>3. S1 AND S2

**Web of Science**<br><br>**Date Searched:** 09/18/2015<br><br>**Number of Results:** 841<br>**After removing Duplicates:** 236	1. TS=(Police OR policing OR "law enforcement" OR officer* OR cop OR cops OR "structural determinant*" OR "structural factor*" OR "social control" OR "criminal law" OR "Social Justice" OR "Civil Rights" OR justice OR "civil right*" OR "Human Rights" OR "crime prevention")<br><br>2. TS=("sex work*" OR "sexual work*" OR sexwork* OR prostitut* OR "commercial sex*" OR "transactional sex*" OR "trading sex*" OR "traded sex*" OR "sex transaction*" OR "sexual transaction*" OR "exchanging sex*" OR "exchanged sex*" OR "sexual favor*" OR "trade sex*" OR "exchange sex*")<br><br>3. #1 AND #2
**International Bibliography of the Social Sciences (IBSS)**<br><br>**Date Searched:** 09/18/2015<br><br>**Number of Results:** 642<br>**After removing Duplicates:** 324	1. SU.EXACT.EXPLODE("Police" OR "Investigations (Law Enforcement)" OR "Law Enforcement" OR "Criminal Law" OR "Social Control" OR "Social Justice" OR "Civil Rights" OR "Human Rights" OR "Womens Rights" OR "Crime Prevention" OR "Criminal Justice" OR "Criminal Justice Policy") OR ti(police* OR policing OR "law enforcement" OR officer* OR cop OR cops OR "structural determinant*" OR "structural factor*" OR "social control" OR "criminal law" OR justice OR "civil right*" OR "crime prevention") OR ab(police* OR policing OR "law enforcement" OR officer* OR cop OR cops OR "structural determinant*" OR "structural factor*" OR "social control" OR "criminal law" OR justice OR "civil right*" OR "crime prevention")<br><br>2. SU.EXACT.EXPLODE("Prostitution") OR ti("sex work*" OR "sexual work*" OR sexwork* OR prostitut* OR "commercial sex*" OR "transactional sex*" OR

	"trading sex*" OR "traded sex*" OR "sex transaction*" OR "sexual transaction*" OR "exchanging sex*" OR "exchanged sex*" OR "sexual favor*" OR "trade sex*" OR "exchange sex*") OR ab("sex work*" OR "sexual work*" OR sexwork* OR prostitut* OR "commercial sex*" OR "transactional sex*" OR "trading sex*" OR "traded sex*" OR "sex transaction*" OR "sexual transaction*" OR "exchanging sex*" OR "exchanged sex*" OR "sexual favor*" OR "trade sex*" OR "exchange sex*")<br>3. 1 AND 3
**Global Health Library (WHO Regional databases**<br><br>**Date Searched:** 09/18/2015<br><br>**Number of Results:** AIM(4), WPRIM(38), IMEMR(27), IMSEAR(27)<br><br>**After removing Duplicates:** AIM(4), WPRIM(37), IMEMR(27), IMSEAR(24)	1. (Police OR policing OR "law enforcement" OR officer OR officers OR cop OR cops OR "criminal law" OR Justice OR "Civil Rights" OR "civil right" OR "Human Rights" OR "crime prevention") AND (Prostitute OR prostitutes OR prostitution OR sex OR sexual OR sexwork OR sexworker OR sexworkers)
**LILACS**<br><br>**Date Searched:** 09/18/2015<br><br>**Number of Results:** 63<br>**After removing Duplicates:** 55	1. (Police OR policing OR MH:I01.880.604.646$ OR MH:M01.526.373.750$ OR MH:M01.526.760$ OR "law enforcement" OR MH:I01.880.604.594$ OR officer* OR cop OR cops OR "structural determinant" OR "structural determinants" OR "structural factor" OR "structural factors" OR "social control" OR MH:I01.655.500$ OR MH:I01.880.604.825$ OR MH:N03.623.500$ OR "criminal law" OR MH:I01.198.290$ OR MH:I01.880.604.583.100$ OR MH:SP9.120.080.010$ OR justice OR MH:I01.880.604.473.700$ OR

	MH:K01.559.411.756.750$ OR MH:K01.752.566.479.830.750$ OR MH:N03.706.437.700$ OR "civil right" OR "civil rights" OR MH:I01.880.604.473.352$ OR MH:N03.706.437.352$ OR "Human Rights" OR MH:I01.880.604.473$ OR MH:N03.706.437$ OR MH:SP9.020$ OR "crime prevention") **AND** ("sex work" OR "sex worker" OR "sex workers" OR "sexual work" OR "sexual worker" OR "sexual workers" OR sexwork OR sexworker OR sexworkers OR MH:M01.776$ OR prostitut* OR MH:F01.145.802.790$ OR MH:I01.880.735.679$ OR "commercial sex" OR "commercial sexual" OR "transactional sex" OR "transactional sexual" OR "trading sex" OR "trading sexual" OR "traded sex" OR "traded sexual" OR "sex transaction" OR "sexual transaction" OR "exchanging sex" OR "exchanged sex" OR "exchanged sexual" OR "sexual favor" OR "sexual favors" OR "trade sex" OR "exchange sex")
**IndMed**<br><br>**Date Searched:** 09/18/2015<br><br>**Number of Results:** 45<br>**After removing Duplicates:** 35	1. Police OR policing OR law enforcement OR officer OR officers OR cop OR cops OR criminal law OR Justice OR Civil Rights OR civil right OR Human Rights OR crime prevention **AND** Prostitute OR prostitutes OR prostitution OR sex OR sexual OR sexwork OR sexworker OR sexworkers
**WHOLIS**<br><br>**Date Searched:** 09/18/2015<br><br>**Number of Results:** 9<br>**After removing Duplicates:** 8	1. "Police" OR policing OR "law enforcement" OR officer OR officers OR cop OR cops OR "criminal law" OR justice OR "civil right" OR "civil rights" OR "Human Rights" OR "crime prevention" OR "social control" **AND** "sex work" OR "sex worker" OR "sex workers" OR "sexual work" OR "sexual worker" OR "sexual workers" OR sexwork OR sexworker OR sexworkers OR prostitute OR prostitutes OR prostitution OR "commercial sex" OR "transactional sex"

Standardized Data Abstraction Form

1. Author
2. Title
3. Year
4. City, Country
5. Settings Legal Approach to sex work
6. Target Population
    a. Cisgender women sex workers (including Injection Drug Users)
    b. Transgender women sex workers (including Injection Drug Users)
7. Sex Work Locations
    a. Multiple locations (e.g., brothels, street corners)
    b. Street-Based Only
8. Study Design and Dates
9. Sample Size
10. Policing Measures
    a. Extra-Legal Police Practices
    b. Legal Police Practices
11. HIV or STI outcome measures
12. HIV/STI sexual risk behaviors
13. Quantitative results
14. Summary of Intervention(s)
15. Notes for authors
